# p62 Overexpression Promotes Bone Metastasis of Lung Adenocarcinoma out of LC3-Dependent Autophagy

**DOI:** 10.3389/fonc.2021.609548

**Published:** 2021-05-21

**Authors:** Dongqi Li, Chuanchun He, Fan Ye, En Ye, Hao He, Gong Chen, Jing Zhang

**Affiliations:** ^1^ Department of Orthopaedics, Bone and Soft Tissue Tumors Research Center of Yunnan Province, The Third Affiliated Hospital of Kunming Medical University, Tumor Hospital of Yunnan Province, Kunming, China; ^2^ Department of Pathology, The Third Affiliated Hospital of Kunming Medical University, Tumor Hospital of Yunnan Province, Kunming, China

**Keywords:** p62/sequestosome 1, bone metastasis, lung cancer, prognosis, autophagy, LC3

## Abstract

p62 protein has been implicated in bone metastasis and is a multifunctional adaptor protein usually correlated with autophagy. Herein, we investigated p62 expression and its prognostic significance in bone metastasis of lung adenocarcinoma, and analyzed whether the mechanism involved depends on autophagy. mRNA and protein expression of p62, LC3B and Beclin 1 were detected by reverse transcription-quantitative PCR and western blotting, respectively, in fresh bone metastasis tissues (n=6 cases) and normal cancellous bone tissues (n=3 cases). The association between p62 and LC3B expression and patient prognosis was subsequently analyzed in 62 paraffin-embedded bone metastasis specimens by immunohistochemistry assay. Small interfering RNA (siRNA) was employed to downregulate p62 expression in SPC-A-1 and A549 cells. Cell proliferation and migration ability were tested by CCK8, CCF and Transwell assays respectively. Autophagy was induced by Rapamycin or inhibited by Atg 7 knockout/Chloroquine in A549 cells and p62 and LC3II/I expression were analyzed. After subcutaneous inoculation or intracardial injection of A549 cells into nude mice, the effect of p62 downregulation *in vivo* was analyzed by histopathological examination. The results showed that p62, LC3B and Beclin 1 mRNA and protein were all overexpressed in bone metastasis tissues (all P<0.01). Patient samples with high p62 expression levels were significantly associated with more bone lesions (>3), shorter overall survival rates and shorter progression free survival rates compared with patients having lower p62 expression (P=0.014, P=0.003, P=0.048, respectively). Cox regression analysis identified p62 expression as an independent prognostic indicator of overall survival of patients with bone metastasis (P=0.007). *In vitro* p62 downregulation inhibited SPC-A-1 and A549 cells migration but had no effect on cell proliferation. After autophagy induction or inhibition, p62 expression involved in autophagy flux and changed inconsistently according to the switch of LC3I to LC3II in different autophagy conditions. *In vivo* p62 downregulation had no effect on growth of subcutaneous tumor. Lung or bone metastasis lesion was not found in all mice model. These findings suggested that p62 overexpression promotes tumor cell invasion out of LC3-dependent autophagy, which could be used a potential prognostic biomarker and therapeutic target for bone metastasis of lung adenocarcinoma.

## Introduction

Lung cancer presents the most morbidity and mortality among malignant tumors in China, and lung adenocarcinoma accounts for about 39.7% of cases (1). In recent years, the treatment of lung cancer has made great progress, but once distant metastases occur, the 5-year survival rate drops below 5% (2). About 30-65% of cases will experience different degrees of bone metastasis, followed by severe pain, hypercalcemia, pathological fractures, nerve or spinal cord compression, and other bone related adverse events, the quality of life is seriously decreased (3, 4). Recent studies have shown that tumor cell proliferation and bone metabolism disorders may be two key factors causing bone metastasis, but the key regulatory factors involved are not clear (5).

p62 (also known as sequestosome-1, SQSTM-1 or A170) is a multifunctional adaptor protein, which is generally considered to be a key player in autophagy similar to LC3 and Beclin 1 proteins (6). p62 serves as a link between LC3 protein and ubiquitinated substrates, which incorporate into the completed autophagosome and are degraded in autolysosomes, thus serving as an index of autophagic degradation (7). p62 is highly expressed in many solid tumors and is closely related to tumor proliferation, invasion and metastasis (8, 9). Furthermore, p62 protein is a key regulator of cellular bone metabolism (10). p62 gene mutations are considered to be the main cause of Paget’s disease of bone, which is a skeletal disorder characterized by excessive activation of osteoclasts (11). In primary bone tumors like osteosarcoma (12), giant cell tumor of bone (13), and myeloma (14), p62 overexpression promotes tumor cell invasion and activation of osteoclasts. But its role in bone metastasis is unknown.

In a previous review, we speculated that p62 proteins might be an emerging regulator of bone metastasis (15). The aim of this study was to investigate p62 expression and its prognostic significance in bone metastasis of lung adenocarcinoma, and to analyze whether the related mechanism depends on autophagy.

## Materials and Methods

### Tissue Specimens and Patients

For RT-qPCR and western blotting assays, 6 cases of fresh bone metastasis tissues from lung adenocarcinoma and 3 cases of normal cancellous bone tissues (from amputation limbs) were collected during surgery between May 2017 and July 2018. In addition, 62 paraffin-embedded specimens of bone metastasis tissues were collected between January 2015 and December 2018 for immunohistochemical testing. All cases were histologically and clinically diagnosed at The Third Affiliated Hospital of Kunming Medical University, Tumor Hospital of Yunnan Province (China). The median follow−up time of the patients was 10 months (ranging from 3−26 months). The protocol for the present study was approved by the Medical Institutional and Clinical Research Ethics Committee of Tumor Hospital of Yunnan Province. All patients included in the present study provided informed verbal consent for participation in the study.

### RT−qPCR

Total RNA was extracted using TRIzol (Thermo Fisher Scientific, Inc., USA) from fresh bone metastasis tissues. Real-time quantitative polymerase reaction (RT−qPCR) was performed using the All−in−One™ First−Strand cDNA Synthesis kit (GeneCopoeia, Inc., USA). The temperature protocol was as follows: 37°C for 15 min, 50°C for 5 min, 98°C for 5 min. The primer sequences used for qPCR of p62 and GAPDH were as follows: p62 forward, 5’-CTGGGACTGAGAAGGCTCAC−3’ and reverse, 5’-GCAGCTGATGGTTTGGAAAT-3’; and GAPDH forward, 5’-CTTAGCACCCCTGGCCAAG-3’ and reverse, 5’-ATGTTCTGGAGAGCCCCG-3’. RT-qPCR sequencing was performed using the SYBR Green Master with Rox kit (GeneCopoeia, Inc., USA). The reaction conditions for qPCR were 95°C for 10 min, followed by 35 cycles of 95°C for 15 sec, 60°C for 30 sec and 72°C for 30 sec. The mRNA expression levels in each group were quantified using the 2^-ΔΔCq^ method (16).

### Western Blot Assay

Fresh bone metastasis tissues were harvested using RIPA lysis buffer (Beyotime Institute of Biotechnology, Inc., CHN). Protein concentrations were measured using the bicinchoninic acid protein assay kit (Beyotime Institute of Biotechnology, Inc., CHN). The total protein of each specimen (30 mg/lane) was separated by SDS-PAGE (10% gels), and then transferred onto a polyvinylidene difluoride membrane (EMD Millipore, Inc., USA). B-actin was used as a loading control. The membrane was blocked with 5% bovine serum albumin (BSA, Beijing Solarbio Science & Technology Co., Ltd., CHN) at room temperature for 40 min, and subsequently incubated with mouse anti-p62 (1:1,500; cat. no. ab56416; Abcam), rabbit anti- LC3B (1:1,000; cat. no. ab48394; Abcam, Inc., UK), rabbit anti-Beclin1 (1:1,000; cat. no. ab207612; Abcam, Inc., UK) and mouse anti-β-actin (1:5,000; cat. no. ab6276; Abcam, Inc., UK) primary antibodies overnight at 4°C. The membranes were then incubated with peroxidase-conjugated goat anti-mouse IgG (1:20,000; cat. no. A4416; Sigma-Aldrich; Merck KGaA, Inc., GER) at room temperature for 1 h. Protein bands were visualized using SuperSignal™ West Femto Maximum Sensitivity Substrate reagents (Thermo Fisher Scientific, Inc., USA). The relative gray value of the immune reactive bands was compared using ImageJ software (version 1.46, National Institutes of Health, USA).

### Immunohistochemistry Assay

In brief, bone metastasis tissues were fixed in 10% formaldehyde for 12 h at room temperature. Then paraffin-embedded specimens of bone metastasis tissues were cut into 4-μm sections and baked at 65°C for 30 min. The sections were washed with xylene and rehydrated with 70, 80, 90 and 100% graded ethanol solutions. Tissue sections were submerged for 2 min in an EDTA buffer at 95°C and 90 kPa for antigen retrieval. Subsequently, the sections were treated with 3% hydrogen peroxide in methanol, followed by incubation with 1% rabbit serum albumin (Cell Signaling Technology, Inc., GER) at room temperature for 10 min. The specimens were incubated overnight at 4°C with an anti-p62 antibody (1:800; cat. no. 16177S; Cell Signaling Technology, Inc., GER) or anti- LC3B (1:500; cat. no. ab48394; Abcam., UK). The specimens were then incubated with SignalStain^®^ Boost IHC Detection Reagent (1:1000; cat. no. 8114P; Cell Signaling Technology, Inc., GER) at 37°C for 30 min. The degree of immunostaining of sections was reviewed by light microscope and scored by two independent pathologists. p62 and LC3B staining was scored semi-quantitatively as negative (<10% positively stained cells; score 0), weak (10–25% positively stained cells; score 1), moderate (26–50% positively stained cells; score 2), or strong (more than 50% positively stained cells; score 3). For statistical analysis, scores 0 and 1 together were considered low expression, while scores 2 and 3 together were considered high expression (17).

### Cell Culture and Cell Transfection

SPC-A-1 and A549 cells were purchased from the American Type Culture Collection (ATCC, Rockville, USA). All cells were cultured in Dulbecco’s modified Eagle’s medium (DMEM) (Gibco, Grand Island, USA) supplemented with 10% fetal bovine serum, 100 U/ml penicillin and 100 µg/ml streptomycin (BioWest, Nuaillé, FRA), at 37°C in a humidified atmosphere containing 5% CO_2_. The medium is replaced every 48h. Small interfering RNA (siRNA) targeting p62 and negative control scrambled siRNA were both designed and synthesized by GeneCopoeia Co., Ltd, USA (siRNA-p62, cat. no. HSH021660-LVRU6GP; scrambled siRNA-p62, cat. no. SHCTR001-LVRU6GP). The sequences of the siRNAs were as follows: siRNA-p62, 5’-CCATCCAGTATTCAAAGCATC-3’ and scrambled siRNA, 5’-gcttcgcgccgtagtctta-3’. Atg7 knockout (Atg-/-) by siRNA was purchased from Cell Signaling Technology, Inc., GER (cat. no. #6604). Transfections of SPC-A-1 and A549 cells with siRNA (50 nm) were performed using a Lipofectamine^®^ 3000 kit (Gibco; Thermo Fisher Scientific, Inc., USA), according to the manufacturer’s protocols. Cell density was 10^6^ cells/25 cm dishes. Puromycin (1 µg/ml) was used to kill any cells that were not successfully transfected. A549 cells silenced for p62 or Atg7 were constructed. Subsequent experimentation was conducted after transfection for 48 h.

### Cell Proliferation Assay

Transfected and control SPC-A-1 and A549 cells were plated in 96-well plates in DMEM with 10% FBS (both Gibco; Thermo Fisher Scientific, Inc., USA) at a density of 5000 cells/well. To quantify cell viability, cultures were stained after 24, 48, and 72 hours; 10 μl Cell Counting Kit-8 (CCK-8, Dojindo Molecular Technologies, Inc., JPN) working solution was then added into the wells for 2 h at 37°C according to the manufacturer’s instructions, after which the absorbance was measured at 450 nm using an Epoch Multi-Volume Spectrophotometer system (BioTek Instruments, Inc., USA). Cell colony formation (CCF) assay was conducted as follows. Briefly, transfected and control A549 cells were seeded in 12-well plates at a density of 500 cells per well and cultured with RPMI-1640 containing 10% FBS (Gibco, Grand Island, USA). After 8–10 days of culture, supernatants were discarded, and cells were washed with PBS, fixed with 4% paraformaldehyde for 30 min, and stained with 1% crystal violet (Solarbio, Beijing, China) for 20 min and then counted. The colony numbers were quantified using ImageJ software. Cell survival was calculated relative to that of control cells.

### Transwell Migration Assay

Cells (1 × 10^5^), suspended in DMEM containing 0.1% BSA were added to the top of the Boyden chamber (EMD Millipore, Inc., USA) at 37°C for 2h. The lower chamber contained 10% serum-supplemented medium. After incubation for 24 h at 37°C, a Transwell chamber (EMD Millipore, Inc., USA) was used to determine cell migration ability. A total of 1 × 10^5^ siRNA-p62 transfected A549 cells were suspended in DMEM containing 10% FBS at a density of 5000 cells/well. Cells were subsequently placed onto the top of each chamber. Medium containing 10% FBS was added to the bottom of the chamber. The cells were incubated for 24 h, and then cells on the upside of the membrane were wiped off to remove the non-migrated cells. Cells that had migrated to the underside of the membrane were stained with crystal violet at room temperature for 20 min and visualized under Nikon Eclipse TE2000-U microscope. A total of 4 random fields (magnification, ×100) were scanned and analyzed using the aforementioned ImageJ software.

### GFP-LC3B Expression Assay

pmCherry-EGFP-LC3B-h plasmid was purchased from Ke Lei Biological Technology Co., Ltd, CHN (cat. no. kl-zl-0999). SPC-A-1 and A549 cells were placed on coverslips and transfected with pmCherry-EGFP-LC3B particles (30 viral particles per cell) using the Lipofectamine^®^ 3000 kit (Gibco; Thermo Fisher Scientific, Inc., USA), according to the manufacturer’s protocols. After 18 h, cells were treated with DMSO or D-limonene for 30 min and then fixed with formalin solution, containing 4% paraformaldehyde. Coverslips were mounted with ProLong^®^ Gold antifade mountant containing 4′, 6-diamidino-2-phenylindole, DAPI (Molecular Probes) to visualize nuclei. Images were acquired by confocal microscopy using a 63× objective (Leica TCS SPE Confocal System, Leica Microsystems, GER).

### Experiment *In Vivo*

All animal experiments were approved by the Institutional Animal Care and Use Committee at the Kunming Medical University. Male BABL/c nude mice (6 weeks of age, 20g–22 g) were purchased from Shanghai SLAC Laboratory Animal Co. (Shanghai, China). Mice were maintained in a specific pathogen-free facility. To assess the effect of p62 downregulation on tumor growth *in vivo*, the stably transfected A549 cells (shR-P62 and scrambled control) were 10^6^/100 µl injected subcutaneously into the opposite flanks of mice (n = 5). Tumor volumes were measured with a micrometer caliper and calculated as (length × width^2^)/2 every 2 days from the fifth day after injection. At the end of the experiment, mice were anesthetized with isoflurane and euthanized on the 14th day, and tumor tissues were collected, photographed and weighed. To assess the effect of p62 downregulation on tumor metastasis, intracardial injection of cells as described above (n = 5), the mice were dissected on the 28th day to search metastasis lesion in lung and bone by histopathological examination.

### Statistics Analysis

Statistical analyses of immunohistochemistry assay were performed using the SPSS 17.0 software package (SPSS, Inc., USA). The significance of the differences between groups was estimated using the χ^2^ test. The significance of the correlation between groups was estimated using Pearson correlation analysis. Progression-free or overall survival curves were plotted according to the Kaplan-Meier method, and compared using the log-rank test. The significance of survival variables was evaluated using a multivariate Cox proportional hazards regression analysis. Statistical graphs were drawn using GraphPad Prism v.6.0 (GraphPad Software, Inc., USA). The differences of p62, LC3B, and Beclin1 mRNA and protein expression levels between two groups of fresh bone metastasis tissues and normal cancellous bone tissues were tested with a Student’s t-test. The differences of p62 and LC3II/I protein expression levels, cell proliferation rates and migration cell numbers between multiple groups were tested by one-way ANOVA with post-hoc Tukey’s test. All data are presented as the mean ± standard deviation of three independent experiments. P<0.05 was considered to indicate a statistically significant difference.

## Results

### p62 and Autophagic Protein Was Overexpressed in Bone Metastasis Tissues

**Figure 1:**
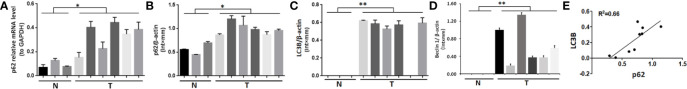
Expression of p62, LC3B and Beclin 1 in bone metastasis tissues of lung adenocarcinoma. **(A, B)** Both p62 mRNA and protein expression levels were higher in tumor tissues (T) than in normal cancellous bone tissues (N). **(C)** LC3B and **(D)** Beclin 1 protein expression levels were higher in tumor tissues (T) than in normal cancellous bone tissues (N). **(E)** Positive correlation between p62 protein expression and LC3B protein expression (P=0.005). T, bone metastasis tissues; N, normal cancellous bone tissues. ^*^P < 0.05, ^**^P < 0.01.

In 6 cases of fresh bone metastasis tissues and 3 cases of normal cancellous bone tissues, p62 mRNA and protein were overexpressed in tumor tissues compared with normal tissues, as determined by RT-qPCR and western blot assays, respectively (**Figures 1A, B**). Both LC3B protein and Beclin 1 protein are overexpressed in tumor tissues compared with normal tissues (**Figures 1C, D**). The western blot results was provides in **Figure S1** for quantification. There was a positive correlation between p62 protein and LC3B protein expression (R2 = 0.66, P = 0.005, **Figure 1E**). There was no correlation between p62 protein and beclin1 protein expression, LC3II/I protein and beclin1 protein expression (**Figure S2**). Thus, our subsequent experiments mainly focus on p62 and LC3B expression to explore the role of p62 in autophagy.

### p62 Overexpression Was Associated With Poor Prognosis of Patients With Bone Metastasis of Lung Adenocarcinoma

**Figure 2:**
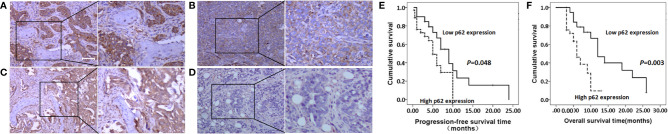
p62 and LC3B protein expression as determined by immunohistochemistry assay. **(A, B)** High p62 (A, scale bar 100µm) and LC3B **(B)** expression at magnification ×100 (left) and ×200 (right). **(C, D)** Low p62 **(C)** and LC3B **(D)** expression at magnification ×100 (left) and ×200 (right). **(E, F)** High p62 expression was associated with shorter progression-free survival rates **(E)** and overall survival rates **(F)** in patients with bone metastasis of lung adenocarcinoma.

As shown in **Figures 2A–D**, and as evidenced by the mostly cytoplasmic and limited cytomembrane staining, p62 and LC3B protein were widely expressed in lung adenocarcinoma cells. To assess the association between p62/LC3B expression levels and clinicopathological features, the tumor specimens were classified into a high expression level group and a low expression level group (**Table 1**). High p62 expression levels were significantly associated with greater than 3 bone metastasis lesions (P = 0.014), and not with age (P = 0.636), sex (P = 0.914), or pathological fractures (P = 0.35). Furthermore, patients in the high p62 expression group had shorter overall survival rates and shorter progression free survival rates compared with patients in the low p62 expression group (P=0.003, P = 0.048, respectively; **Figures 2E, F**). In three serial specimens of lung tumor, lymph node metastasis and bone metastasis tissues, p62 staining gradually increased (**Figures S3**). But high LC3B expression levels showed no association with any clinicopathological characteristics (**Table 1**). Correlation analysis showed that there was no correlation between p62 protein and LC3B protein (R^2^ = 0.06, P = 0.15). Cox regression analysis identified p62 expression as an independent prognostic indicator of overall survival in patients with bone metastasis of lung adenocarcinoma (P = 0.007, **Table 2**).

**Table 1:** Association of p62 and LC3B protein expression with clinicopathological features of patients with lung adenocarcinoma bone metastasis.

	p62		P	LC3B		P
	Low expression	High expression		Low expression	High expression	
Age						
(years)	52.68±8.71	53.92±10.51	0.636	52.51±10.229	55.13±9.172	0.316
Sex						
Male	27	13	0.914	27	13	0.230
Female	12	10		12	10	
Number of bone metastasis						
≤3	16	16	0.014	17	13	0.235
>3	6	24		22	10	
Pathologic fracture						
Yes	14	22	0.350	22	14	0.471
No	8	18		17	9	

**Table 2:** Cox regression analysis of overall survival in 62 patients with lung adenocarcinoma bone metastasis.

Clinicopathological features	B	SE	Wald	df	Sig.	Exp (B)	95.0% CI for Exp (B)	
							Lower	Upper
p62	-1.317	0.490	7.229	1	0.007	0.268	0.103	0.700
LC3B	-0.615	0.365	2.838	1	0.092	0.541	0.264	1.106
Age	0.030	0.021	1.967	1	0.161	1.031	0.988	1.075
Sex	0.190	0.363	0.273	1	0.601	1.209	0.594	2.460
Number of bone metastasis	0.377	0.393	0.919	1	0.338	1.458	0.675	3.150
Pathologic fracture	0.076	0.403	0.036	1	0.850	1.079	0.490	2.379

B, regression coefficient; SE, standard error; Wald, χ^2^ value; df, degree of freedom; Sig, significance; Exp(B), odds ratio; CI, confidence interval.

### p62 Downregulation Inhibited Migration but Not Proliferation of Lung Adenocarcinoma Cells

**Figure 3:**
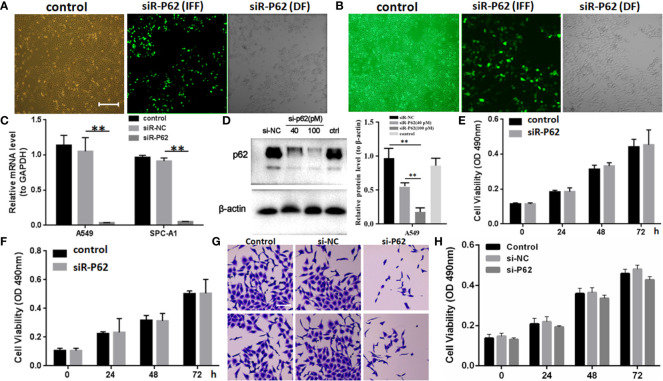
p62 downregulation using small interfering RNA transfection did not inhibit tumor proliferation in SPC-A-1 and A549 cells. **(A)** Small interfering p62 RNA (siR-p62) plasmid was successfully transfected in A549 cells. The left control indicates untreated tumor cells. The middle siR-p62 (IFF) indicates small interfering p62 RNA-transfected tumor cells in immunofluorescence field. The right siR-p62 (DF) indicates small interfering p62 RNA-transfected tumor cells in dark field (scale bar 200µm). **(B)** siR-p62 plasmid was successfully transfected in SPC-A-1 cells. **(C)** After 48h p62 mRNA expression level was downregulated in siR-p62 transfected cells than scrambled control cells (siR-NC). **(D)** p62 protein expression level was downregulated in siR-p62 transfected cells than scrambled control cells (siR-NC). **(E, F)** The difference in proliferation between siR-p62 transfected group and scrambled control group (siR-NC) of A549 **(E)** and SPC-A-1 **(F)** cells was found to be not significant between 0 h to 72 h. **(G, H)** Cell colony formation assay showed the difference in proliferation between siR-p62 transfected group and scrambled control group (siR-NC) of A549 cells was not significant between 0 h to 72 h (scale bar 50µm). control, untreated A549 or SPC-A-1 cells; siR-p62, small interfering p62 RNA transfected cells; siR-NC, siR-p62 scrambled control; IFF, immunofluorescence field; DF, dark field; ^**^P < 0.01.

**Figure 4:**
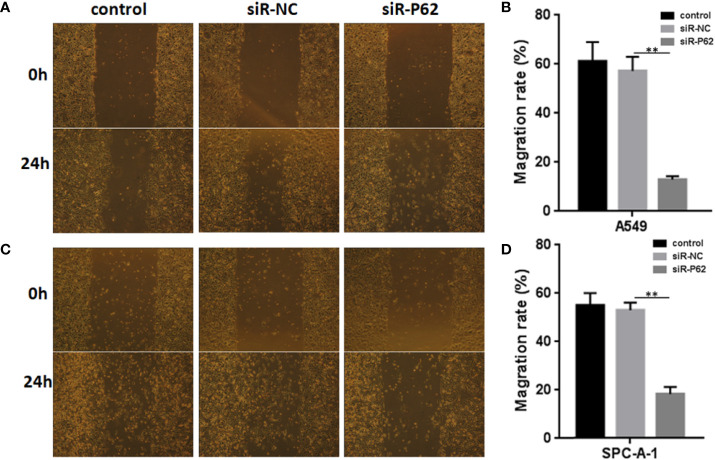
p62 downregulation inhibits the migration of A549 and SPC-A-1 cells. **(A)** Migration of A549 cells with different p62 expression. **(B)** p62 downregulation inhibited the migration of A549 cells. **(C)** Migration of SPC-A-1 cells with different p62 expression. **(D)** p62 downregulation inhibited the migration of SPC-A-1 cells. control, untreated A549 or SPC-A-1 cells; siR-p62, small interfering p62 RNA transfected cells; siR-NC, siR-p62 scrambled control. ^**^P < 0.01.

**Figure 5:**
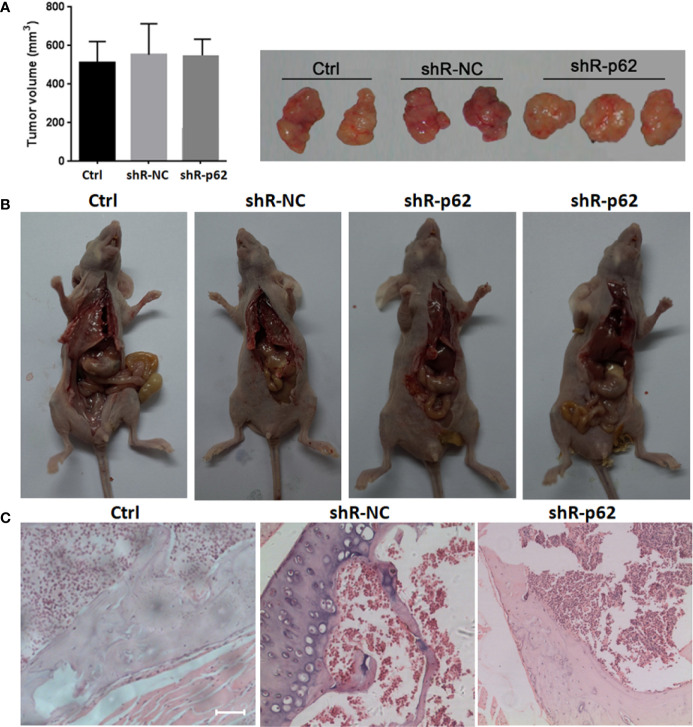
*In vivo* p62 downregulation had no effect on growth of subcutaneous tumor. **(A)** The difference of tumor volumes between silenced p62 group (n = 3), scrambled control group (n = 2) and control group (n = 2) was not statistically significant. **(B)** All mice model was dissected on the 28th day, and lung or bone metastasis lesion was not found. **(C)** Bone metastasis lesion of limb or spine was not found under microscope (scale bar 200µm). Ctrl, untreated A549 cells; shR-p62, stably transfected A549 cells; shR-NC, shR-p62 scrambled control.

The expression of p62 was downregulated by small interfering RNA transfection in SPC-A-1 and A549 cells. After 48h, p62 mRNA expression was inhibited as assessed by RT-qPCR assay (**Figures 3A–C**). Western blot assay also showed p62 protein expression was downregulated in siR-p62 transfected cells than scrambled control cells (**Figure 3D**). The differences between the silenced p62 gene cells (siRp62 group) and the scrambled control (siR-NC) group were analyzed. The difference in proliferation measured by relative optical density (OD) values between the two groups was not statistically significant between 0h to 72h (**Figures 3E, F**). The same results showed p62 downregulation had no effect on cell colony formation by CCF assay (**Figures 3G, H**). However, the downregulation of p62 led to the inhibition of migration of SPC-A-1 and A549 cells (**Figure 4**). *In vivo* assays, the volumes of subcutaneous tumor were 602.4 mm^3^, 550.9 mm^3^ in control group (n = 2), 608.3 mm^3^, 560.8 mm^3^ in scrambled control group (n = 2) and 596.4 mm^3^, 564.8 mm^3^, 598.2 mm^3^ in silenced p62 group (n = 3). The difference was not statistically significant between three groups (**Figure 5A**). Lung or bone metastasis lesion was not found in all mice model by histopathological examination (**Figures 5B, C**).

### p62 Expression Was Independent of Autophagy

**Figure 6:**
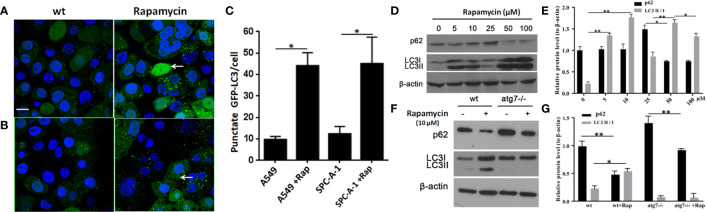
Changes in detection of p62 and LC3 II/I protein upon induction or inhibition of autophagy. **(A)** In untreated wild type A549 cells, punctate GFP-LC3 (white arrow) was rare (left image) but increased significantly after exposure to rapamycin (10 μM/L) for 24 h (right image) by GFP-LC3 immunofluorescence microscopy. **(B)** Similar to wild type SPC-A-1 cells, punctate GFP-LC3 increased slightly after treatment with rapamycin. **(C)** Punctate GFP-LC3 positive cells were increased after rapamycin exposure both in A549 and SPC-A-1 cells. **(D)** p62 and LC3 II/I protein expression upon different concentrations of exposure to rapamycin for 24h. **(E)** Densitometric analysis of protein bands showing LC3 II/I protein expression increased from 5μM/L to 10μM/L and decreased from 50 μM/L to 100 μM/L, but p62 protein expression changes were not obvious. **(F)** Changes in detection of p62 and LC3 II/I protein upon autophagic gene 7 knockout (Atg7-/-). **(G)** Densitometric analysis of protein bands showing LC3 II/I protein expression almost disappeared after Atg7-/- knockout, but p62 protein expression decreased obviously both after autophagy induction by Rapamycin and autophagy inhibition by Atg7-/- knockout. Scale bar was 20 μm. wt, wild type A549 or SPC-A-1 cells; Rap, Rapamycin. ^*^P < 0.05, ^**^P < 0.01.

**Figure 7:**
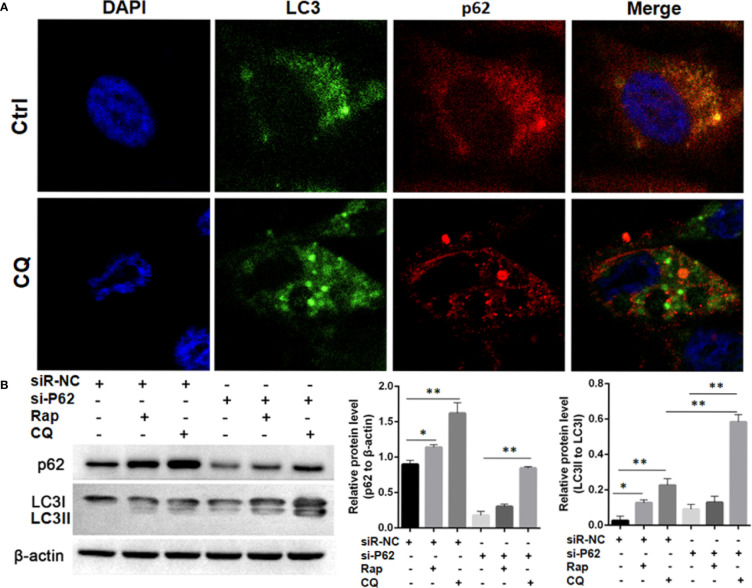
Autophagy flux detection upon treating with Chloroquine (CQ) or Rapamycin (Rap) in wild type and p62 silencing A549 cells. **(A)** Double immunofluorescent staining of p62 and LC3 protein in A549 cells treated with CQ (20μM/L for 48h). Punctate GFP-LC3 emits green fluorescence, and punctuate mCherry-p62 emits red fluorescence. In merged panel p62 protein only partly colocalized with LC3 protein. **(B)** Changes of p62 and LC3 protein was detected upon Rap or CQ in different A549 cells. Significant switch of LC3 I to LC3 II was detected in different A549 cells treated with CQ or Rap, which demonstrates that autophagy flux is activated. Meanwhile, increased p62 expression is induced instead of degradation as autophagy substrate. In p62 silencing A549 cells, Significant switch of LC3 I to LC3 II was detected upon treating with CQ but not with Rap. Ctrl, untreated A549 cells; siR-p62, small interfering p62 RNA transfected cells; siR-NC, siR-p62 scrambled control; CQ, Chloroquine; Rap, Rapamycin. ^*^P < 0.05, ^**^P < 0.01.

In SPC-A-1 and A549 cells, autophagy was induced by rapamycin, which increased the number of positive GFP-LC3 cells as detected by confocal microscopy (**Figures 6A–C**). We used A549 cells for subsequent experiments because of the more obvious autophagic puncta in these cells. The consistent results showed LC3II/I expression level was increased after Rapamycin treatment by western blot assay. Furthermore, there was not a clear correlation between the increases in LC3II/I expression and the decrease in p62 expression (**Figures 6D, E**). Following autophagy inhibition by Atg 7 gene knockout, LC3II/I expression almost disappeared but p62 expression decreased slightly following treatment with Rapamycin (**Figures 6F, G**). In A549 cells treated with lysosomal proteolysis inhibitors, p62 expression increased but only partly colocalized with LC3 protein (**Figure 7A**). As classic substrate of autophagy degradation, p62 expression changed inconsistently according to the switch of LC3I to LC3II in different autophagy conditions (**Figure 7B**). These results demonstrated that p62 expression involved in autophagy flux, which was independent of LC3 expression. In brief, p62 expression might function out of LC3-dependent autophagy.

## Discussion

According to the “seed and soil” hypothesis, bone metastasis is dependent on the interactions between tumor cells and the bone microenvironment including the fenestrated capillaries in bone, bone matrix, and cells in the bone marrow (BM) stroma, such as osteoblasts and osteoclasts (18). Identification of key regulators between tumor cells and the bone microenvironment could clarify molecular mechanism involved and will improve clinical treatment of bone metastasis. Our results showed there was a higher mRNA and protein expression of p62 in bone metastasis of lung adenocarcinoma compared with normal cancellous bone tissue, suggesting p62 may be involved in tumor formation or metastasis during gene transcription and protein translation. p62 is not only overexpressed in early-stage lung cancer (19), but it is also associated with poor prognosis of patients with lung adenocarcinoma (17, 20). Our results were consistent with previous research (21). Furthermore, we found p62 expression gradually increased when transitioning from lung tumor, lymph node metastasis and bone metastasis tissues, which suggested that p62 protein might promote tumor invasion of lung adenocarcinoma. We also confirmed p62 downregulation inhibited lung adenocarcinoma cells invasion *in vitro*. In addition, p62 overexpression also showed a stronger association with bone metastasis lesions, which also suggested p62 overexpression played a key role in the bone microenvironment and metabolism (10). The ability of p62 to modulate tumor cells and osteoclasts suggested that it may be a feasible target for bone metastasis and especially for osteolytic metastasis (15).

p62 is considered a key autophagy-associated protein like LC3B and Beclin1 (7). Targeting autophagy is a promising therapeutic strategy to overcome bone tumor and metastasis (22, 23). Our results showed that LC3B and Beclin1 were also overexpressed in bone metastasis tissues, but only LC3B expression had positive correlation with p62 expression by western blot assay. Nonetheless, there was no obvious correlation between p62 expression and LC3B expression by immunohistochemistry, which was not consistent with western blot assay. So we continue to focus on both p62 and LC3B in next studies.

We further verified whether p62 protein could regulate bone metastasis of lung adenocarcinoma by participating in autophagy. p62 and LC3B proteins are the most common markers of autophagic activity. The LC3B protein precursor is processed to excise the carboxyl terminal to produce LC3I protein, which in turn covalently binds to phospholipids on the autophagosome membrane to generate LC3II protein (24). As a substrate for autophagy ubiquitination, p62 protein binds LC3II protein and translocates to the autophagosome for degradation. Therefore, the expression of p62 protein and LC3II/I protein are generally negatively correlated, which dynamically reflects the dynamic changes in autophagy (7). In our study, p62 expression involved autophagy flux activation, but p62 protein did not vary along with changes in LC3II/I protein in different autophagy conditions, which suggests that p62 could function out of LC3-dependent autophagy. Previous study revealed that oligomerized p62 targeted to the autophagosome formation site independent of LC3 (25). A recent study showed that p62 protein expression was not associated with autophagy under specific conditions (26), while p62 protein has also been shown to promote tumor cell survival by activation of the NF-κb pathway (27). The latest study revealed that p62 expression might target PD-L1, and p62 signaling axis could be useful to suppress the EGFR-TKI-resistant lung cancer (28). Thus, p62 may play versatile role in different cells or in different microenvironments (6).

*In vivo* assay our data showed p62 downregulation did not inhibit tumor growth, which was consistent with the data *in vitro*. But no metastasis lesion was found in mice model, which did not support the results *in vitro*. The reasons may include the amount of samples was small. We will repeat the experiment and change other highly metastatic lung cancer HARA-B, 95d cells in future. Another reason maybe that p62 regulates metastasis in different ways (not overexpression), like inducing Epithelial-Mesenchymal Transition (29). This was a preliminary study, next we will foucs on p62 signaling of promoting metastasis beside autophagy pathway.

In conclusion, our findings confirmed that the overexpression of the autophagic protein p62 promotes bone metastasis of lung adenocarcinoma, although the associated mechanism may be out of LC3-dependent autophagy. p62 could be used a potential prognostic biomarker and therapeutic target for bone metastasis of lung adenocarcinoma. New promising research investigating a p62 vaccine treatment received good response in advanced solid tumors (30, 31) and neurodegenerative disease (32). We propose that this new therapeutic strategy may improve clinical treatment of bone metastasis of lung adenocarcinoma.

## Data Availability Statement

The raw data supporting the conclusions of this article will be made available by the authors, without undue reservation, to any qualified researcher.

## Ethics Statement 

The studies involving human participants were reviewed and approved by Medical Institutional and Clinical Research Ethics Committee of Yunnan Tumor Hospital of China. Written informed consent for participation was not required for this study in accordance with the national legislation and the institutional requirements. The animal study was reviewed and approved by The Institutional Animal Care and Use Committee at the Kunming Medical University.

## Author Contributions

JZ designed this research and revised the manuscript. DL and CH performed the western blot analysis, RT−qPCR assays and cell experiments, and wrote the manuscript. FY performed the p62 and atg7 siRNA assays. EY performed immunohistochemistry assays and HH analyzed the data. GC collected the experiment tissues and followed up the patients. All authors contributed to the article and approved the submitted version.

## Funding

This work was supported by the National Nature Science Foundation of China (grant no. 81760486), and Science and Technology Plan of Yunnan province (grant no. 2018FB133).

## Conflict of Interest

The authors declare that the research was conducted in the absence of any commercial or financial relationships that could be construed as a potential conflict of interest.

## Supplementary Material

Supplementary Figure 1 Expression of p62, LC3B and Beclin 1 in bone metastasis tissues of lung adenocarcinoma by western blot assay.

Supplementary Figure 2 There was no correlation between p62 protein and beclin1 protein expression. There was no correlation between LC3II/I protein and beclin1 protein expression.

Supplementary Figure 3 p62 staining became more and more intense in tumor tissues from lung adenocarcinoma, lymph node metastasis to bone metastasis by immunohistochemistry assay. **(A)** Faint staining in lung adenocarcinoma tissues at magnification ×200 (left, scale bar 100µm) and ×400 (right). **(B)** Moderate staining in lymph node metastasis tissues at magnifications of ×200 (left) and ×400 (right). **(C)** Strong staining in bone metastasis tissues at magnifications of ×200 (left) and ×400 (right).
