# Supplementary figures and images for: p62 Overexpression Promotes Bone Metastasis of Lung Adenocarcinoma out of LC3-Dependent Autophagy
Source: Front Oncol. 2021 May 21;11:609548. doi: 10.3389/fonc.2021.609548 (PMC8175982; doi:10.3389/fonc.2021.609548)

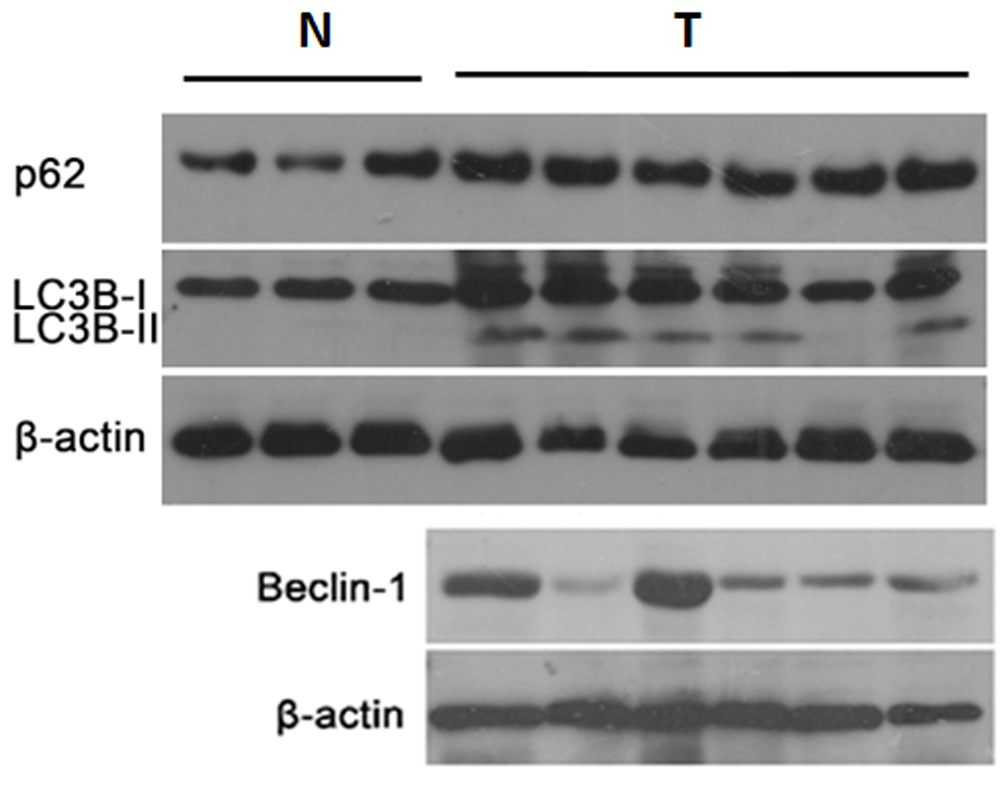

Supplement: Supplementary Figure 1 — Expression of p62, LC3B and Beclin 1 in bone metastasis tissues of lung adenocarcinoma by western blot assay. [file Image_1.tif]

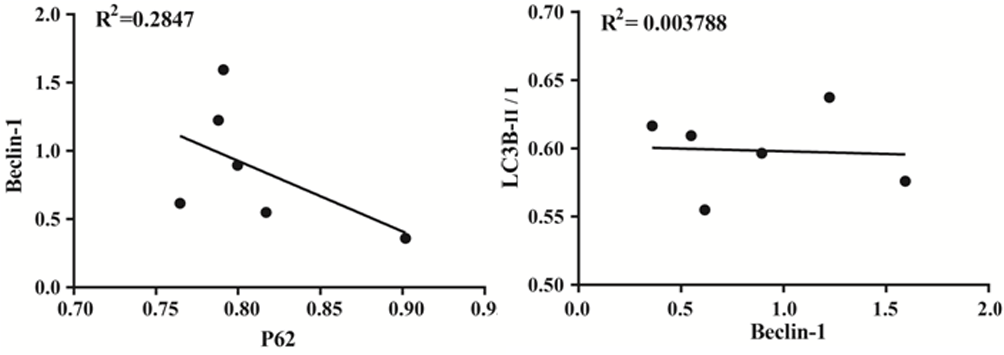

Supplement: Supplementary Figure 2 — There was no correlation between p62 protein and beclin1 protein expression. There was no correlation between LC3II/I protein and beclin1 protein expression. [file Image_2.tif]

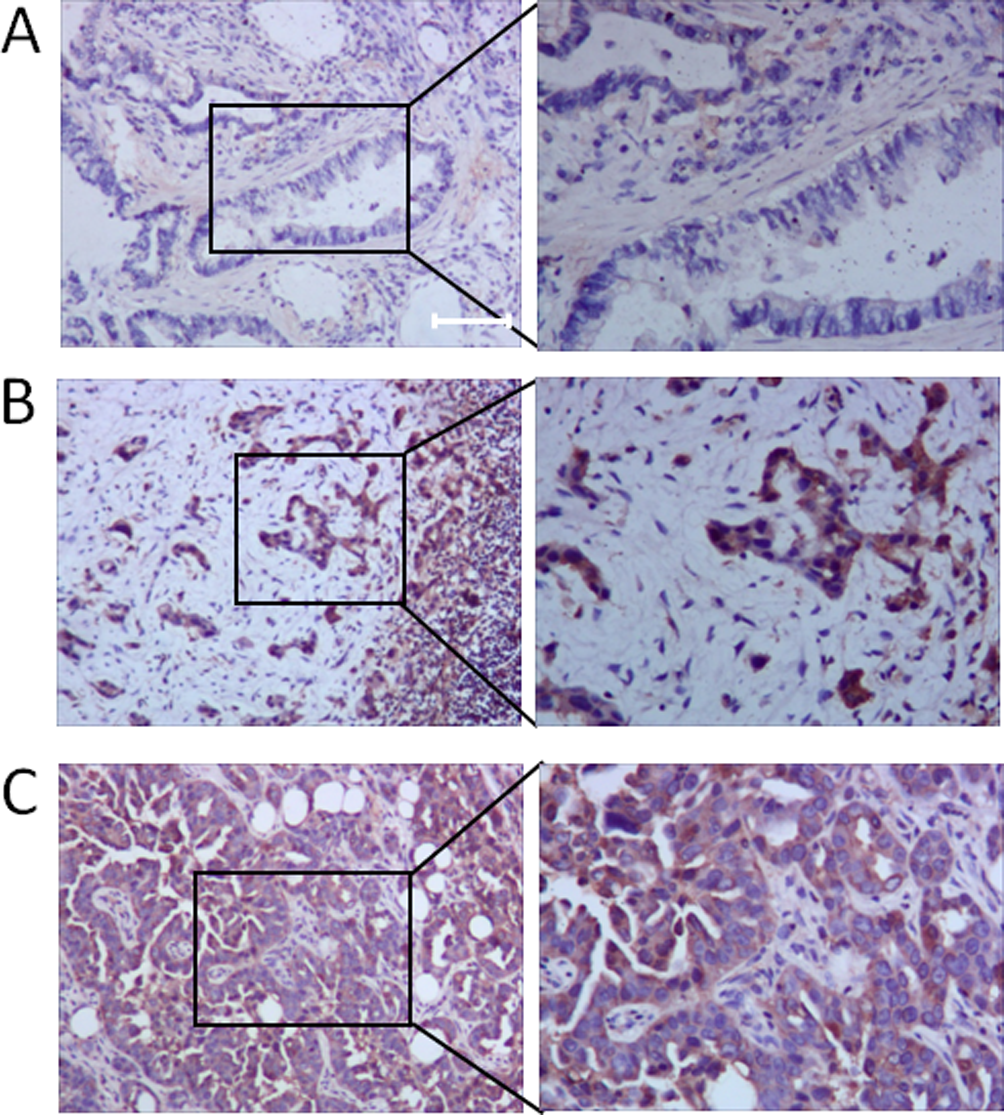

Supplement: Supplementary Figure 3 — p62 staining became more and more intense in tumor tissues from lung adenocarcinoma, lymph node metastasis to bone metastasis by immunohistochemistry assay. (A) Faint staining in lung adenocarcinoma tissues at magnification ×200 (left, scale bar 100µm) and ×400 (right). (B) Moderate staining in lymph node metastasis tissues at magnifications of ×200 (left) and ×400 (right). (C) Strong staining in bone metastasis tissues at magnifications of ×200 (left) and ×400 (right). [file Image_3.tif]
